# Expression of Concern: Targetting an LncRNA P5848-ENO1 axis inhibits tumor growth in hepatocellular carcinoma

**DOI:** 10.1042/BSR-20180896_EOC

**Published:** 2020-08-10

**Keywords:** ENO1, Hepatocellular carcinoma (HCC), LncRNA P5848, Therapeutic target

The Editorial Office has been made aware of potential issues surrounding the scientific validity of this paper, hence has issued an expression of concern to notify readers whilst the Editorial Office investigates.

